# Epitranscriptomic Analysis of m6A Methylome After Peripheral Nerve Injury

**DOI:** 10.3389/fgene.2021.686000

**Published:** 2021-07-09

**Authors:** Lei Zhang, Dingyu Hao, Pengyi Ma, Boyuan Ma, Jia Qin, Guangyuan Tian, Zihao Liu, Xianhu Zhou

**Affiliations:** Department of Orthopedics, Tianjin Medical University General Hospital, Tianjin, China

**Keywords:** peripheral nerve, axon regeneration, m6A, RNA methylation, autophagy

## Abstract

N6-methyladenosine (m6A) is one of the most plentiful internal RNA modifications, especially in eukaryotic messenger RNA (mRNA), which plays pivotal roles in the regulation of mRNA life cycle and nerve development. However, the mRNA m6A methylation pattern in peripheral nervous injury (PNI) has not been investigated. In this study, sciatic nerve samples were collected from 7 days after sciatic nerve injury (SNI) and control rats. Quantitative real-time PCR demonstrated that m6A-related methyltransferase/demethylase genes were remarkably upregulated in SNI group compared with control group. Methylated RNA immunoprecipitation sequencing (MeRIP-seq) was performed to reveal the m6A methylation landscape. The results showed that 4,014 m6A peaks were significantly altered, including 2,144 upregulated and 1,870 downregulated m6A peaks, which were corresponded to 1,858 genes. Moreover, 919 differentially expressed genes were identified by the conjoint analysis of MeRIP-seq and RNA-seq. GO and KEGG pathway analyses were performed to determine the biological functions and signaling pathways of the m6A-modified genes. Notably, these genes were mainly related to the immune system process, cell activation, and nervous system development in GO analysis. KEGG pathway analysis revealed that these genes were involved in the cell cycle, B cell receptor signaling pathway, axon guidance pathway, and calcium signaling pathway. Furthermore, the m6A methylation and protein expression levels of autophagy-related gene (*Atg7*) were increased, together with the activation of autophagy. These findings shed some light on the epigenetic regulation of gene expression, which may provide a new opinion to promote functional recovery after PNI.

## Introduction

Peripheral nerve repair following injury is a complicated process. The distal stump of injured nerve undergoes Wallerian degeneration due to the loss of nutrition and energy support (Waller, 1851; Stoll et al., 2002). Neuronal apoptosis/necrosis and axon disintegration may induce the dedifferentiation and reprogramming of Schwann cells (Arthur-Farraj et al., 2017; Clements et al., 2017). The injury-induced Schwann cells can proliferate to form Bungner’s bands that establish connections between broken stumps and guide the direction of axon regeneration (Waller, 1851). Schwann cells also inhibit the production of myelin protein and remove myelin debris, which contain axon growth inhibitory proteins (e.g., myelin-associated glycoprotein) (Shen et al., 1998). At the same time, Schwann cells secrete various trophic factors that accelerate axon regeneration coincided with the recruitment of immune cells (neutrophils, macrophages, and T lymphocytes) to the nerve injury sites (Gaudet et al., 2011; Richner et al., 2014). Hematogenous macrophages transport across the high permeability blood–nerve barrier to phagocytose myelin debris and enhance distal nerve regeneration (Chen et al., 2015). Apart from the above, Schwann cells activate autophagy to relieve local pressure and balance the nerve microenvironment during axonal regeneration (Pereira et al., 2012). On the 7th day after peripheral nerve injury (PNI), the number and proliferative activity of activated Schwann cells attain the highest level (Zhou et al., 2017). However, the recovery of motor and sensory functions is limited in some patients with severe PNI, and the molecular mechanisms underlying this phenomenon are still unclear.

The roles of epigenetic modifications in the peripheral nervous system are being actively studied, especially histone acetylation (Puttagunta et al., 2014), and DNA methylation (Loh et al., 2017). It has been proven that these epigenetic changes can promote the transcriptional activation of multiple regeneration-associated genes. However, the m6A modification in axon regeneration remains to be characterized. Similar to other epigenetic modifications, m6A methylation is also dynamic and reversible (Füllgrabe et al., 2014; Zhao et al., 2017; Weng et al., 2018). The formation of m6A is regulated by both methyltransferases (WTAP, METTL3, and METTL14; termed as “writers”) and demethylases (FTO and ALKBH5; termed as “erasers”) (Yeo and O’Rahilly, 2012; Zheng et al., 2013; Yue et al., 2015), and then m6A-recognizing proteins (YTHDF1/2, IGF2BP1, and HNRNPA2B1; termed as “readers”) bind to the specific site to mediate its function (Weng et al., 2018). Some studies have demonstrated that m6A methylation affects almost every stage in the messenger RNA (mRNA) cycle by altering RNA structure, regulating maturation, promoting translation, and accelerating mRNA decay (Zhao et al., 2017).

In this study, we first assessed the expression levels of methyltransferase and demethylase genes in sciatic nerve injury (SNI) and control groups. Then, we compared the m6A-tagged transcription profiles before and after SNI *via* methylated RNA immunoprecipitation sequencing (MeRIP-seq) and identified differentially methylated peaks. After that, the data were combined with RNA sequencing (RNA-seq) to further analyze the relationship between mRNA expression and m6A methylation. Finally, we found that the mRNA m6A methylation and protein expression levels of *Atg7* were increased after SNI. ATG7 acts as the hub of microtubule-associated protein light chain 3 (LC3)-phosphatidylethanolamine and ATG12, thereby facilitating the expansion of autophagosomal membranes (Feng et al., 2015). This provides us with a new theoretical basis for PNI treatment.

## Materials and Methods

### Animal Model of Sciatic Nerve Injury

Female Wistar rats (100–120 g, 4 weeks old) were randomly divided into control and SNI groups at different time points. The animal model of SNI was constructed as described previously (Zhou et al., 2017). We collected sciatic nerve samples (about 1 cm) at the center of the injury as well as from the control rats, and then frozen at −80°C until further analysis. All animals were maintained under a standard condition (12:12-h light/dark cycle), with unlimited access to food and water. The experimental procedures involving animals were conducted in accordance with the Institutional Animal Care guidelines of the Radiation Medicine Chinese Academy of Medical Sciences and were approved by the Ethics Committee of the Radiation Medicine Chinese Academy of Medical Sciences.

### RNA Isolation and qRT-PCR

The mRNA expression levels of m6A writers and erasers were detected by quantitative real-time PCR (qRT-PCR). Total RNA was isolated from the sciatic nerve tissues in control (uninjured) and SNI (7 days after injury) groups using a TRIzol Reagent (15596026, Invitrogen). The purity and quantity of RNA samples were assessed by NanoDrop ND-1000 instrument. cDNA was prepared by reverse transcription using a FastKing RT kit with gDNase (KR116, Tiangen, China). QRT-PCR was performed using the Ultra SYBR Mixture (CW0957, Cwbio, China) according to the manufacturer’s specifications. GAPDH was used as an internal control. Each experiment was repeated at least three times, and all data were presented as mean ± standard deviation (SD). The primer sequences are shown in Table 1.

**TABLE 1:** Primers used for qRT-PCR analysis.

**Gene name**	**Primer**	**Sequence**
GAPDH	Forward	GACATGCCGCCTGGAGAAAC
	Reverse	AGCCCAGGATGCCCTTTAGT
ALKBH5	Forward	ACGGCCTCAGGACATCAAAG
	Reverse	AAGCATAGCTGGGTGGCAAT
WTAP	Forward	GGTACAAGATGACCAACGAAGA
	Reverse	ATTAACTCATCCCGTGCCATAA
METTL3	Forward	GCACTTCAGACGGATTATCAAC
	Reverse	TCATAGTGGACATACTTGCAGG
METTL14	Forward	GCCGACAGATTTGAAGAATACC
	Reverse	CACATCAAACTTGGGTGTCAAT
FTO	Forward	CAGAGATCCCGATACGTGGC
	Reverse	CTGTGAGCCAGCCAAAACAC

### MeRIP-seq and RNA-seq

The sequencing experiments were conducted at Cloud-seq Biotech Inc. (Shanghai, China). First, we collected and quantified total RNA from the sciatic nerve tissues of control (uninjured) and SNI (7 days after injury) groups. Then, Arraystar Seq-StarTMpoly(A) mRNA Isolation Kit (Arraystar, MD, United States) was used to extract poly(A) mRNA from total RNA. The tubes with 1 μg/μL RNA and fragmentation buffer (metal-ion) were incubated at 70°C for 6 min in a preheated thermal cycler block. Subsequently, 30 μL of 3 M sodium acetate (NaOAc), 1 μL of glycoBlue, and 750 μL volumes of 100% ethanol were added, mixed and kept at −80°C overnight. After centrifugation and pellet resuspension, RNA fragmentation was conducted by running 0.5 μg of RNA through 1.5% (wt/v) agarose gel. The fragmented RNA was immunoprecipitated with magnetic beads containing m6A antibody (1 μL m6A antibody in 50 μL 1 × IP buffer) using the GenSeq^TM^ m6A RNA IP kit (GenSeq Inc., China). The input samples without immunoprecipitation were used as a control group. The following reagents were used for elution and purification in sequence: 1 × IP buffer, LB Buffer, HS Buffer, RLT Buffer, 100% and 75% ethanol.

MeRIP-seq libraries were constructed using the NEBNext^®^ Ultra II Directional RNA Library Prep Kit (New England Biolabs, Inc., United States). For RNA-seq, total RNA (1 μg) was used to remove the rRNAs *via* Ribo-Zero rRNA Removal Kit (Illumina, San Diego, CA, United States). RNA-seq libraries were constructed using the TruSeq Stranded Total RNA Library Prep Kit (Illumina, San Diego, CA, United States). Then, both libraries were controlled and evaluated with the Bio Analyzer 2100 system (Agilent Technologies, United States). The 10 pM libraries were denatured as single-stranded DNA molecules and amplified *in situ* as clusters. MeRIP-seq and RNA-seq procedures were carried out on an Illumina NovaSeq 6000 instrument with 150-bp paired-end reads. The quality control metrics of the sequencing data are listed in Supplementary Table 1.

### Sequencing Data Analysis

For MeRIP-seq data analysis, paired-end reads were conducted *via* Illumina NovaSeq 6000 sequencer and were quality checked with Q30. Cutadapt software (v1.9.3) was used to remove 3′ adaptor-trimming and low-quality reads (Martin, 2011). Clean reads of all libraries were mapped to the reference genome (Rat5) *via* Hisat2 software (v2.0.4) (Kim et al., 2015). Peaks with m6A modification were detected by MACS software (Zhang et al., 2008). Differentially methylated sites were identified by diffReps and visualized through IGV software (Shen et al., 2013). The peaks recognized by both software and overlapped with two or more exons were filtered out and annotated by homemade scripts. MEME software was used to perform motif-based sequence analysis (Bailey, 2011). GO and KEGG pathway analyses were conducted to identify the biological functions and signaling pathways of differentially m6A-modified genes.

For mRNA sequencing data analysis, the paired-end reads from Illumina NovaSeq 6000 sequencer were quality checked with Q30, and the low-quality reads were removed by Cutadapt software (v1.9.3) (Martin, 2011). Then, the high-quality clean reads from MeRIP-seq were aligned to the reference genome (Rat5) *via* Hisat2. Cuffdiff software (part of Cufflinks) was used to calculate the gene level FPKM (fragments per kilobase of exon per million fragments mapped reads) as the expression profiles of mRNA. Differentially expressed genes were identified based on the fold-change and *p*-value calculated from FPKM values (Trapnell et al., 2010). GO and KEGG pathway analyses were performed to assess the biological functions and signaling pathways of differentially expressed genes.

### Gene-Specific m6A qPCR

Gene-specific m6A qPCR was carried out at Cloud-seq Biotech Inc. First, we obtained and quantified the total RNA from both control (uninjured) and SNI (7 days after injury) groups. Then, metal-ion was used to fragment the extracted RNA to approximately 100 nt in length and reserved one-tenth of fragmented RNA as INPUT control. Next, the fragmented RNA were subjected to immunoprecipitation with m6A antibody-coated magnetic beads. Methylated RNA bound to the magnetic beads was eluted and purified. Finally, the m6A modification of *Atg7* gene was verified by qPCR analysis. The primer sequences are listed in Table 1.

### Western Blot Analysis

The sciatic nerve samples obtained from control (uninjured) and SNI (2, 5, 7, and 10 days after injury) groups were ground using an automatic grinder, and then lysed with RIPA lysis buffer. BCA protein assay kit (P0012, Beyotime) was used to measure the concentration of total protein. An equal amount of protein sample was separated by 10% SDS-PAGE, and then transferred onto polyvinylidene difluoride membranes. After blocking with 5% non-fat milk for 1 h, the membranes were incubated with primary antibody overnight at 4°C. Subsequently, the membranes were incubated with the corresponding secondary antibody for 1 h. Enhanced chemiluminescent substrate (Bio-Rad) was used to visualize the protein bands. The antibodies used for Western blotting were as follows: ATG7 (1:1000, 8558S; Cell Signaling Technology), LC3 (1:1,000, L7543; Sigma-Aldrich), and GAPDH (1:2,000, ab181602; Abcam).

### Transmission Electron Microscopy

The fresh samples of control and SNI (2 days after injury) groups were fixed with 2.5% glutaraldehyde overnight at 4°C and then with OsO4 at 4°C for 1 h. Different concentrations of ethanol solution (50, 70, 80, 90, 95, and 100%) were used for gradient dehydration. The samples were permeated with acetone and Epon embedding agent, sectioned using an ultramicrotome, and stained with lead citrate solution and uranyl acetate saturated in 50% ethanol. Autophagosomes were observed under a JEM-1010 electron microscope (Jeol, Tokyo, Japan) at × 3,000 magnification.

### Statistical Analysis

All statistical analyses were performed using the GraphPad Prism software. The results were presented as mean ± SD. ImageJ software was used to calculate the gray values of Western blot images. Significant differences between two groups were assessed by Student’s *t* test, ANOVA, and *post hoc* analysis. Fisher’s exact test was used for all bioinformatic analyses.

## Results

### Ubiquitous Expression of m6A Writers and Erasers in Sciatic Nerve After Injury

**FIGURE 1:**
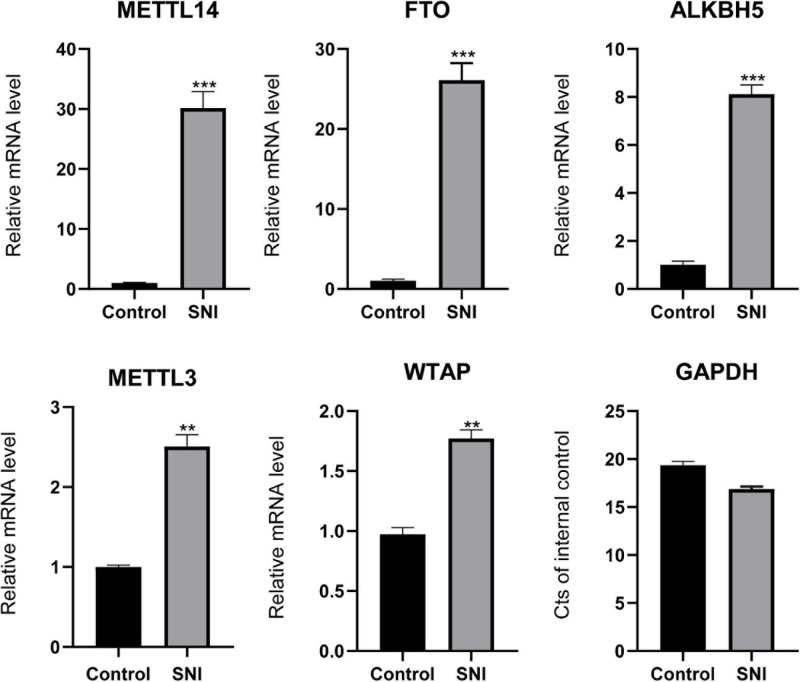
The expression levels of writers and erasers. Quantitative RT-PCR showing the upregulated levels of METTL3, METTL14, WTAP, FTO, and ALKBH5. Among them, the expression levels of METTL14 and FTO were the most significant. GAPDH was used as an internal control. SNI group (*n* = 3); control group (*n* = 3). “**” and “***” indicate *p* < 0.01 and *p* < 0.001, respectively, *via* Student’s *t* test.

To investigate the effects of epigenetic modulators on rat sciatic nerve (7 days after SNI), we determined the expression profiles of m6A writers (or known as methyltransferases; METTL3, METTL14, and WTAP) and erasers (or known as emethylases; FTO and ALKBH5). As shown in Figure 1, the mRNA expression levels of these methyltransferases and demethylases were remarkably higher in SNI group than in control group. Among them, the expression levels of FTO (26.1) and METTL14 (30.1) were more significantly upregulated compared with other key genes. The ubiquitous expression of these genes indicates that m6A plays a prominent role during SNI.

### Transcriptome Profile of m6A Methylation in SNI Samples

**FIGURE 2:**
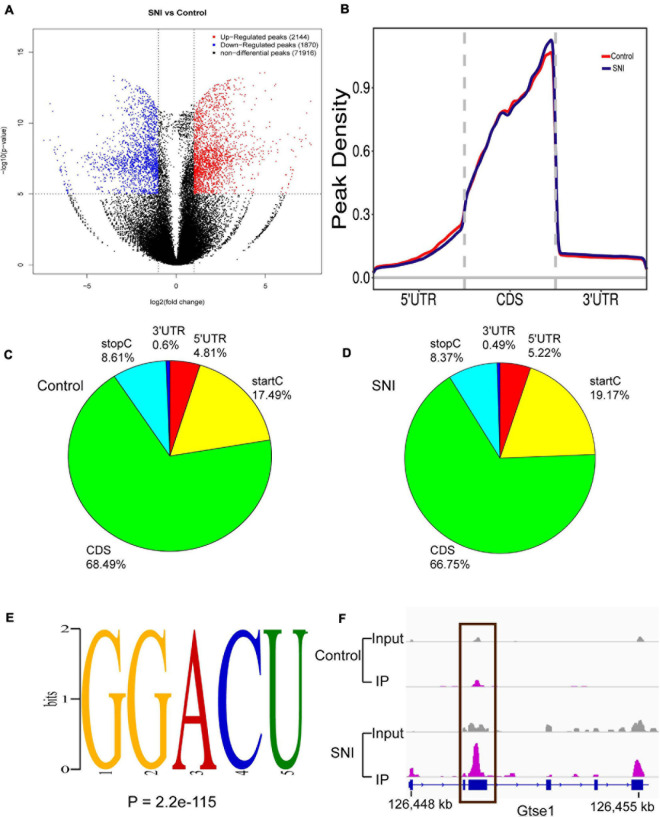
The degree and pattern of m6A modification peaks in SNI and control groups. **(A)** Volcano plots indicating the distribution of differential m6A peaks after SNI. Fold-change ≥ 2.0, *p* < 0.00001, *n* = 3, *via* Fisher’s exact test. **(B)** Priority region and average distribution of m6A peaks. **(C,D)** The distribution of m6A peaks in SNI and control groups, respectively. **(E)** The top motifs enriched across differential m6A peaks. **(F)** Data visualization of the m6A-modified *Gtse1* mRNA.

To analyze the level and distribution of m6A methylation in SNI and control groups, MeRIP-seq was performed to assess the epitranscriptome m6A-seq data (GEO accession number: GSE171866). In total, SNI samples had 2,144 and 1,870 significantly upregulated and downregulated m6A peaks, which corresponded to the transcripts of 1,083 and 775 genes, respectively (fold-change ≥ 2.0 and *p* < 0.00001; Figure 2A). Among them, the top 20 differentially methylated m6A peaks are shown in Table 2, and the remaining m6A peaks can be found in Supplementary Table 2.

**TABLE 2:** The top 20 altered methylated m6A mRNAs.

**Gene name**	**Fold change**	**Regulation**	**Peak start**	**Peak end**	**Peak region**	***p*-value**
Bcl11b	188.6	Up	141083119	141083485	CDS	3.41184E-09
C1qtnf3	185.8	Up	83764121	83764184	stopC	2.90904E-12
RT1-M2	180.8	Up	1330021	1330145	CDS	2.60969E-09
Hmga2	159.7	Up	65477106	65477157	CDS	1.95917E-11
Ly49i9	154.5	Up	212860534	212860641	3UTR	1.23827E-09
Gtse1	154.4	Up	126447843	126447961	startC	7.05159E-10
Slco5a1	132.9	Up	10704800	10705520	5UTR	1.52433E-09
Ccr7	132.1	Up	86863313	86863401	3UTR	4.51785E-09
Klri2	128.5	Up	212206301	212206516	stopC	2.02144E-09
Ttc22	121.6	Up	130071681	130071960	startC	2.23587E-10
Oxtr	170.7	Down	207715424	207715580	stopC	1.67168E-09
Itih6	133.8	Down	37820981	37821720	CDS	1.59935E-09
Obscn	133.7	Down	45231005	45231120	CDS	4.28052E-12
Olr681	120.6	Down	84659858	84660480	CDS	1.47375E-11
Fam160a1	114.3	Down	204333641	204334040	CDS	7.85335E-09
Adamts20	113.7	Down	135185042	135185100	CDS	7.74537E-09
Cxxc4	111	Down	257943781	257944120	5UTR	1.16816E-08
AABR06107100.1	110.3	Down	106884509	106884591	CDS	4.91596E-09
Il17a	107.4	Down	25696864	25696949	5UTR	1.51155E-08
Grip1	100.7	Down	64974137	64974160	CDS	7.01286E-09

We further identified the preferential locations of m6A peaks in mRNA coding regions. The results demonstrated that the peaks were markedly correlated with the coding sequence (CDS), and there were significantly enriched peaks at the beginning of 3′ untranslated region (3′UTRs) and near the stop codon (Figures 2B–D). Motif enrichment analysis indicated that GGACU was the most enriched m6A sites of were in both SNI and control groups (Figure 2E), which provides further proof for the conservativeness of m6A methylation. *Gtse1*, a significantly hypermethylated peak on the CDS, was also detected (Figure 2F). These results strongly suggest that m6A methylation is dynamic and reversible.

### Biological Functions and Signaling Pathways Associated With Differentially Methylated mRNA

**FIGURE 3:**
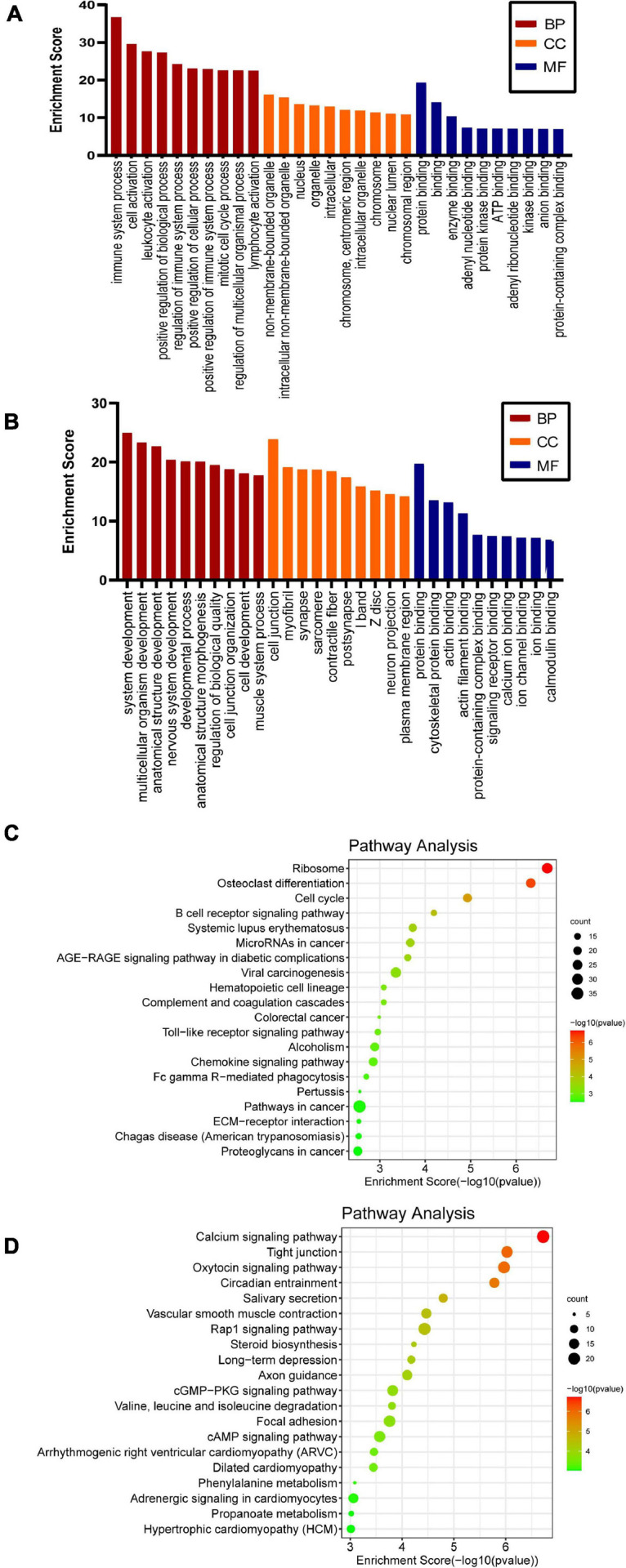
GO and KEGG pathway analyses of the differentially methylated genes. Significantly enriched GO terms of the hypermethylated **(A)** and hypomethylated genes **(B)**. Significantly enriched KEGG pathways of the hypermethylated **(C)** and hypomethylated **(D)** genes.

To clarify the biological functions and signaling pathways of m6A modification during SNI, both GO enrichment and KEGG pathway analyses were carried out. In GO analysis, the biological functions of differentially methylated genes can be classified into three parts: biological processes (BP), cellular component (CC), and molecular function (MF). The top 10 most remarkably enriched BPs, CCs, and MFs of the genes with up/downregulated m6A peaks are listed in Figures 3A,B. Through the KEGG pathway analysis, the crucial pathways of differentially m6A-modified genes were identified. The results showed that the hypermethylated genes were involved in the cell cycle, B cell receptor signaling pathway, and Toll-like receptor (TLR) signaling pathway (Figure 3C). The hypomethylated genes were involved in the calcium signaling pathway, Rap1 signaling pathway, and axon guidance (Figure 3D). This indicates m6A methylation is a potential target to improve sciatic nerve repair after injury.

### Conjoint Analysis of MeRIP-seq and RNA-seq Data

**FIGURE 4:**
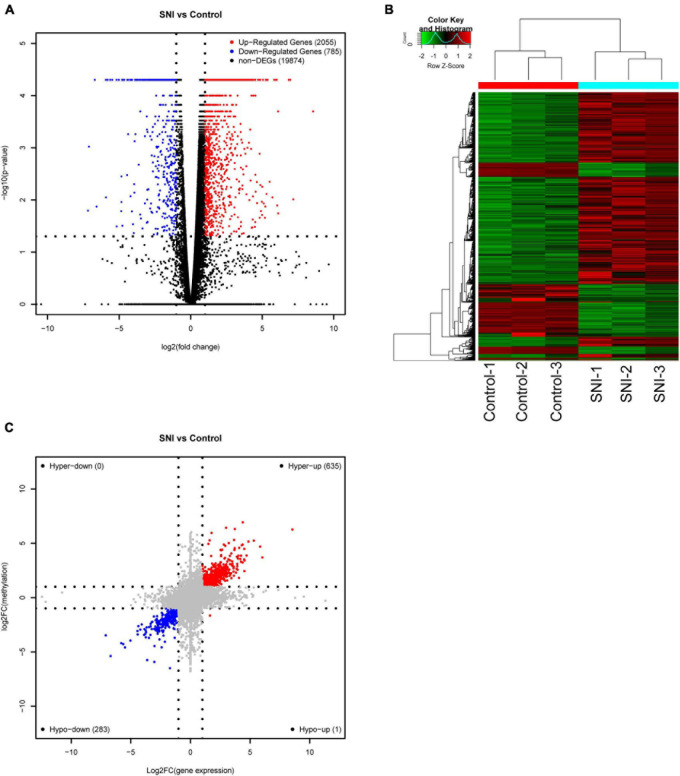
Conjoint analysis of m6A methylation and mRNA expression after SNI. **(A)** Volcano plots and **(B)** Heatmap plots displaying the differentially expressed genes between SNI and control groups. **(C)** Four-quadrant graph showing the association between mRNA m6A methylation and transcriptome level.

To explore the association between gene expression and m6A methylation, RNA-seq data were used to determine the extent of altered mRNA expression (GEO accession number: GSE171866). The differentially expressed transcripts consisted of 2,055 upregulated transcripts and 785 downregulated transcripts (fold-change ≥ 2.0 and *p* < 0.05; Figure 4A). The top 20 differentially expressed genes are listed in Table 3, while the top 10 GO terms and KEGG pathways are presented in Supplementary Figure 2. Cluster analysis was performed to assess the expression patterns of differential methylated genes (Figure 4B). In the light of the conjoint analysis of MeRIP-seq and RNA-seq data, all differentially m6A peaks with differential mRNA levels were divided into four groups (Figure 4C). Notably, all 635 hypermethylated m6A peaks in mRNA transcripts were upregulated, while 283 of the 284 hypomethylated m6A peaks were downregulated in mRNA transcripts. These results suggest that there is a strong correlation between m6A methylation and gene expression.

**TABLE 3:** The top 20 differently expressed mRNAs in SNI based on log2 (fold change).

**Gene name**	**log2 (fold change)**	**Regulation**	**Locus**	**Strand**	***p*-value**
Gpr83	8.55401	Up	chr8:13306229-13316717	+	0.0002
LOC680329	7.17537	Up	chr11:89193034-89194878	+	0.00975
LOC100362684	6.96595	Up	chr5:165588419-165588924	+	0.00005
Igkc	6.8567	Up	chr4:163306241-163309315	+	0.00005
Ccl9	6.12582	Up	chr10:70416899-70421785	−	0.01805
Cd19	6.05504	Up	chr1:204797141-204803557	−	0.01275
Tyrp1	6.02478	Up	chr5:102430673-102464565	+	0.00005
Cd8a	5.97504	Up	chr4:164017632-164021869	+	0.00005
Cd8b	5.72046	Up	chr4:163964374-163980435	+	0.00895
Lilrb4	5.84134	Up	chr1:75149297-75154600	+	0.00005
Myh2	−7.17359	Down	chr10:53467096-53491312	+	0.01595
Mb	−7.10582	Down	chr7:118094390-118101652	−	0.00095
Myh4	−6.7028	Down	chr10:53530952-53553894	+	0.00005
Atp2a1	−6.57491	Down	chr1:204820052-204854008	−	0.0134
Ckm	−5.9313	Down	chr1:81587879-81598112	+	0.00005
Sypl2	−5.92245	Down	chr2:230472383-230487115	−	0.00895
Myh1	−5.80509	Down	chr10:53493996-53530654	+	0.00005
Actn3	−5.62826	Down	chr1:227051530-227067458	−	0.00005
Acta1	−5.48312	Down	chr19:67389900-67392928	−	0.00005
Pgam2	−5.18691	Down	chr14:86736788-86738938	−	0.00005

### The m6A Methylation and Protein Expression Level of *Atg7* Are Increased in SNI Group

**FIGURE 5:**
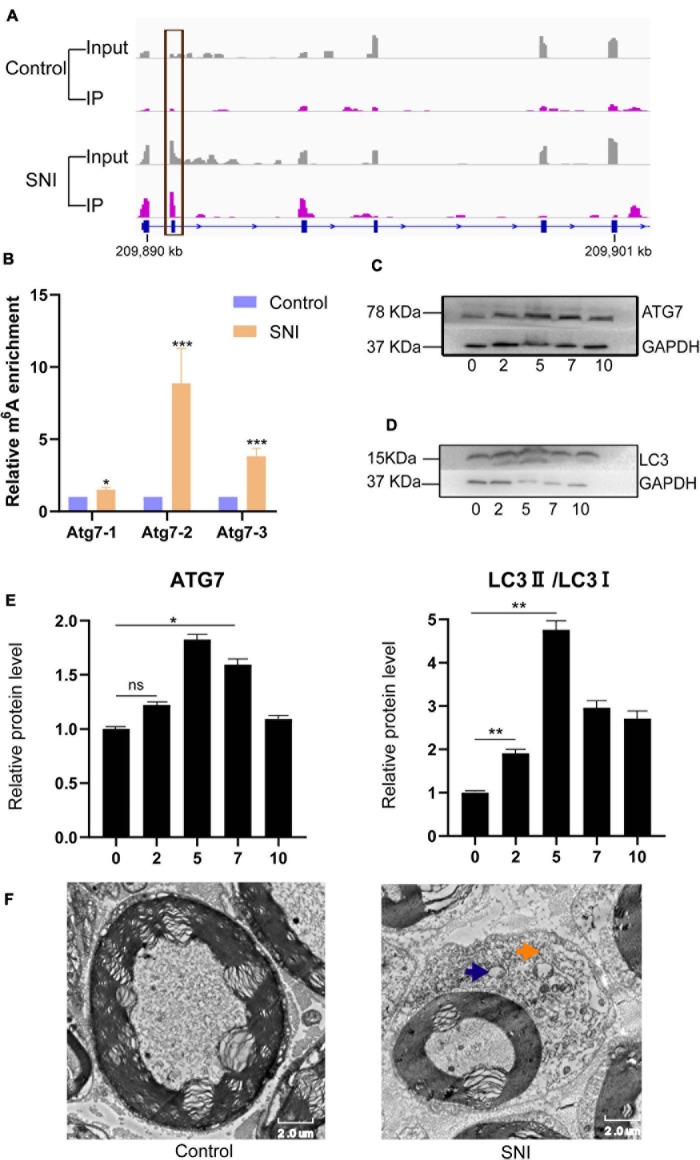
The m6A methylation and protein expression of *Atg7* gene are altered, and autophagy is activated following PNI. **(A,B)** Integrative genomics viewer and gene-specific m6A qPCR demonstrating the increased methylation of *Atg7* gene after SNI. *n* = 3, *via* Student’s *t* test. **(C)** Upregulation of ATG7 protein level. *n* = 3, *via* Western blot analysis. **(D)** Expression level of the autophagy-related marker LC3 is increased. *n* = 3. **(E)** Quantification of Western blots for ATG7 and LC3II/LC3I. *n* = 3, *via* ANOVA and *post hoc* analysis. **(F)** Transmission electron microscopy showing autophagosome (yellow arrow) and lysosome (blue arrow) in the sciatic nerve tissues. “*,” “**,” and “***” indicate *p* < 0.05, *p* < 0.01, and *p* < 0.001, respectively.

Autophagy plays a vital role in the repair of SNI. In this study, we found that the m6A abundance of *Atg7* mRNA was obviously increased after SNI (Figure 5A). To further verify the expression of *Atg7* mRNA methylation, gene-specific m6A qPCR was performed. Interestingly, the result of gene-specific m6A qPCR was consistent with that of m6A-seq (Figure 5B). The protein expression of *Atg7* was also upregulated, indicating m6A methylation may serve as a potential mechanism to regulate autophagy during SNI (Figures 5C,E). In addition, the expression of the autophagy-related marker LC3 was increased (Figures 5D,E), and the autophagosomes were abundantly found in sciatic nerve after injury (Figure 5F). Altogether, our findings reveal a prominent association between the protein expression of *Atg7* and its mRNA methylation.

## Discussion

The roles of m6A modification in neurodevelopment have been verified in these aspects, including learning and memory (Li et al., 2019). Despite these efforts, the epigenetic changes in rat sciatic nerve remain poorly understood. In this study, we obtained a landscape of m6A methylation after SNI *via* MeRIP-Seq. There were 4,014 differentially m6A peaks, corresponding to 2,144 genes, and each gene was associated with at least one or more m6A peaks. The distribution of m6A peaks appeared in the CDS, which was also consistent with other diseases such as cancer (Zhang et al., 2020b), traumatic brain injury (Yu et al., 2020), and preeclampsia (Wang et al., 2020a). After PNI, these m6A peaks preferentially located at the start codons (19.17 vs. 17.49%), and there was a decreasing trend in the CDS (66.75 vs. 68.49%). The motif sequence GGACU displayed remarkably higher proportions than other motifs in both control and SNI groups, as well as in other species (e.g., human and mice). Besides, it is noteworthy that RRACH (where R denotes purine, A stands for m6A, and H represents a non-guanine base) is their consensus sequence (Dominissini et al., 2012; Zhang et al., 2020a).

In addition, we found that the mRNA expression levels of methylase and demethylase genes were upregulated in SNI group compared with control group. GAPDH is generally used as a housekeeping gene for the normalization of gene expression data. However, in our results, the Ct values of GAPDH in SNI and control groups were 16.9 and 19.3, respectively. This might be due to the fact that our samples were derived from rat sciatic nerve tissue. Compared with cells, tissue samples are more difficult to obtain the same Ct values. Some studies have pointed out that the mRNA expression of β-actin is increased after axon injury and may play a certain role in the acceleration of axonal outgrowth (Sántha et al., 2012; Donnelly et al., 2013).

The upregulated expression levels of FTO and METTL14 were more significant compared with other key genes. FTO and METTL14 act as eraser and writer, respectively, which regulate the dynamic levels of m6A RNA methylation. Some studies have suggested that FTO promotes the initiation of autophagy by reducing the mRNA methylation levels of *Atg5*, *Atg7*, and *Ulk1* (Jin et al., 2018; Wang et al., 2020b). Autophagy is activated after PNI to promote nerve regeneration and motor recovery (Huang et al., 2016). However, the methylation level of *Atg7* mRNA was increased in our study, and there were no significant differences in the mRNA methylation patterns of *Atg5* and *Ulk1* between SNI and control groups. In the next step, we will determine which methyltransferase regulates *Atg7* mRNA and construct a conditional gene knockout model to verify its role in PNI. Perhaps that it is a new way to clarify the underlying mechanism of autophagy after PNI.

*Fto* depletion inhibits the length of dendrites and the number of intersections in primary neurons and dynamically regulates the proliferation and differentiation of adult neural stem cells (Cao et al., 2020). Another study reported that a conditional knockout of *Fto* in brain lipid inhibits adult neurogenesis by inducing cell apoptosis (Gao et al., 2020). FTO promotes neuropathic pain by elevating *G9a* expression in primary sensory neurons (Li et al., 2020). *G9a* is also involved in the regulation of autophagy *via* linking to the promoter of *Atg* gene (Talebian et al., 2020). On the other hand, FTO can be bound with a variety of reaction substrates (e.g., m6Am and m1A), suggesting the complexity and functionality of this gene (Wei et al., 2018).

METTL14 plays an important role in the formation of multicomponent methyltransferase complex as a reaction catalyst to activate and promote METTL3 activity (Wang et al., 2017). In the central nervous system, depletion of *Mettl14* prevents the differentiation and maturation of oligodendrocytes, thus resulting in a decreased number of mature cells and hypomyelination (Xu et al., 2020). After PNI, the premyelinating phenotypes of Schwann cells are characterized by neural cell adhesion molecule, glial fibrillary acidic protein, and p75 neurotrophin receptor (Chen et al., 2007). Our results also showed that METTLE4 was upregulated, suggesting that it may have the potential to promote the development, maturation, and regeneration of Schwann cells. Besides, *Mettl14* knockout in cortical neural progenitor cells can slow down the cell-cycle progression. Thus, it is speculated that METTL14 may also affect the development and regeneration of neural progenitor cells after PNI (Yoon et al., 2017).

KEGG pathway analysis revealed that the TLR signaling pathway was regulated by the genes with upregulated m6A modification sites during SNI. TLRs are dependent on pathogen-related molecular patterns to induce activities that stimulate immunity and host defense (Fitzgerald and Kagan, 2020). Recent findings have demonstrated that the expression of TLRs is upregulated in activated Schwann cells after nerve injury, which are involved in Wallerian degeneration and proinflammatory mediator generation, thereby recruiting more immune cells into the nerve injury sites (Thakur et al., 2017). TLR7 and TLR8 participate in neuropathic pain by activating NF-κB and Erk signaling pathways, respectively (Zhang et al., 2018; He et al., 2020). In *Tlr2* knockout mice, thermal hyperalgesia and mechanical allodynia were attenuated compared with wild-type mice (Kim et al., 2007). According to our results, we believe that modulating m6A modifications of the TLR signaling pathway may be a potential therapeutic target for SNI in the near future.

Axon guidance pathway was regulated by the genes with downregulated m6A-modified sites during SNI. These genes included *Ablim1*, *Cxcl12*, *Dpysl5*, *Ephb6*, *L1cam*, *Lrrc4*, *Ntn1*, *Plxnb1*, *Robo3*, *Sema4g*, *Sema5a*, *Sema6d*, *Slit2*, and *Slit3*, all of which could be used as potential targets for regulating axon growth. CXCL12 is primarily responsible for Schwann cell migration and autophagy by regulating PI3K-AKT-mTOR signaling pathway (Gao et al., 2019); L1CAM promotes Schwann cell-neurite interactions and enhance neurite outgrowth (Romano et al., 2015); NTN1 could induce Schwann cell proliferation and migration to form the Büngner bands and extend toward the distal nerve stump (Dun and Parkinson, 2017).

Based on the conjoint analysis of MeRIP-seq and RNA-seq data, 635 hypermethylated m6A genes were upregulated, and 283 of 284 hypomethylated m6A genes were downregulated. There was a significant correlation between m6A methylation and mRNA expression level. Indeed, m6A not only affects mRNA folding and maturation to induce protein expression, but also regulates mRNA decay to curtail its lifetime (Zhao et al., 2017). Both methyltransferase and demethylase genes can determine the m6A status of a random mRNA; and in turn, the m6A-binding proteins can target and categorize the methylated mRNA into different functional groups. For example, YTHDF1 interacts with eIF3 to increase translation efficiency (Wang et al., 2015); while YTHDF2 binds to mRNA decay sites, thereby resulting in mRNA instability and degradation (Wang et al., 2014).

Among the 635 hypermethylated m6A genes, three genes (*Atf3*, *Creb1*, and *Jun*) were associated with functional recovery after PNI. In *Atf3* mutant mice, the axon regeneration ability of DRG neurons is weakened (Gey et al., 2016). Overexpression of *Creb* enhances the regeneration of lesioned dorsal column axons (Gao et al., 2004). The loss of neuron *Jun* decreases the rate of axon regeneration after crushing, and blocks most severe axons from reconnecting to their targets (Ruff et al., 2012). Therefore, it can be inferred that m6A-modified genes play critical roles in the development of peripheral nervous system. However, detailed molecular mechanisms are still unknown, and this aspect deserves careful consideration in the near future.

## Conclusion

This study reveals the m6A RNA methylation landscape *via* high-throughput sequencing, which sheds new light on the pivotal roles of epigenetic changes in SNI. Conjoint analysis of m6A-RIP-seq and RNA-seq indicated the effect of m6A methylation on regulating gene expression. The m6A methylation and protein expression level of *Atg7* were increased, laying the foundation for understanding the epitranscriptomic mechanisms of autophagy. Further experiments should be carried out to verify the differential methylated genes in SNI patients.

## Data Availability Statement

The datasets presented in this study can be found in online repositories. The names of the repository/repositories and accession number(s) can be found below: NCBI Gene Expression Omnibus, accession no: GSE171866.

## Ethics Statement

The animal study was reviewed and approved by Ethics Committee of Radiation Medicine Chinese Academy of Medical Sciences.

## Author Contributions

LZ and DH participated in the design of the research and the writing of the manuscript. PM, BM, and JQ conducted the experiments. GT and ZL analyzed the data. XZ revised the manuscript and were responsible for supervision of study. All authors contributed to the article and approved the submitted version.

## Conflict of Interest

The authors declare that the research was conducted in the absence of any commercial or financial relationships that could be construed as a potential conflict of interest.
