# Supplementary material for: Epitranscriptomic Analysis of m6A Methylome After Peripheral Nerve Injury
Source: Front Genet. 2021 Jul 9;12:686000. doi: 10.3389/fgene.2021.686000 (PMC8301379; doi:10.3389/fgene.2021.686000)
Supplement: Supplementary file 1 [file Table_1.DOCX]

**Table S1. Quality control of m6A-RIP-seq and RNA-seq.**

| **m6A-RIP-seq** | | | | | | | |  |
| --- | --- | --- | --- | --- | --- | --- | --- | --- |
|  |  |  |  |  |  |  |  |  |
| **Sample** | **Group** | **Raw Reads** | **Clean Reads** | **Clean Ratio** | **Mapped Reads** | **Mapped Ratio** | **Q30** |  |
| **CONTROL001** | CONTROL001-Input | 55,387,906 | 55,381,170 | 99.99% | 51,042,371 | 92.17% | 94.32% |  |
|  | CONTROL001-IP | 48,298,492 | 48,197,588 | 99.79% | 41,271,713 | 85.63% | 91.98% |  |
| **CONTROL002** | CONTROL002-Input | 36,546,756 | 36,541,060 | 99.98% | 33,643,553 | 92.07% | 93.89% |  |
|  | CONTROL002-IP | 47,433,448 | 47,286,234 | 99.69% | 41,580,513 | 87.93% | 91.42% |  |
| **CONTROL003** | CONTROL003-Input | 39,116,376 | 39,109,888 | 99.98% | 35,918,075 | 91.84% | 94.34% |  |
|  | CONTROL003-IP | 47,640,322 | 47,502,082 | 99.71% | 41,172,156 | 86.67% | 91.68% |  |
| **SNI001** | SNI001-Input | 48,821,008 | 48,814,742 | 99.99% | 44,536,218 | 91.24% | 93.88% |  |
|  | SNI001-IP | 47,690,346 | 47,575,686 | 99.76% | 40,777,924 | 85.71% | 91.57% |  |
| **SNI002** | SNI002-Input | 50,970,328 | 50,961,440 | 99.98% | 46,732,808 | 91.70% | 93.57% |  |
|  | SNI002-IP | 52,946,878 | 52,872,666 | 99.86% | 45,171,701 | 85.43% | 92.12% |  |
| **SNI003** | SNI003-Input | 49,522,366 | 49,515,340 | 99.99% | 45,626,973 | 92.15% | 94.05% |  |
|  | SNI003-IP | 47,436,980 | 47,322,634 | 99.76% | 40,611,402 | 85.82% | 91.77% |  |

| **RNA-Seq** | | | | | | |  |
| --- | --- | --- | --- | --- | --- | --- | --- |
|  |  |  |  |  |  |  |  |
| **Sample** | **Raw Reads** | **Clean Reads** | **Clean Ratio** | **Mapped Reads** | **Mapped Ratio** | **Q30** |  |
| **CONTROL001** | 48,821,008 | 48,817,620 | 99.99% | 41,022,351 | 84.03% | 93.88% |  |
| **CONTROL002** | 50,970,328 | 50,964,846 | 99.99% | 43,155,751 | 84.68% | 93.57% |  |
| **CONTROL003** | 49,522,366 | 49,518,498 | 99.99% | 42,240,547 | 85.30% | 94.05% |  |
| **SNI001** | 55,387,906 | 55,384,680 | 99.99% | 46,536,813 | 84.02% | 94.32% |  |
| **SNI002** | 36,546,756 | 36,543,622 | 99.99% | 31,066,184 | 85.01% | 93.89% |  |
| **SNI003** | 39,116,376 | 39,112,438 | 99.99% | 33,189,012 | 84.86% | 94.34% |  |
