# Supplementary figures and images for: Epitranscriptomic Analysis of m6A Methylome After Peripheral Nerve Injury
Source: Front Genet. 2021 Jul 9;12:686000. doi: 10.3389/fgene.2021.686000 (PMC8301379; doi:10.3389/fgene.2021.686000)

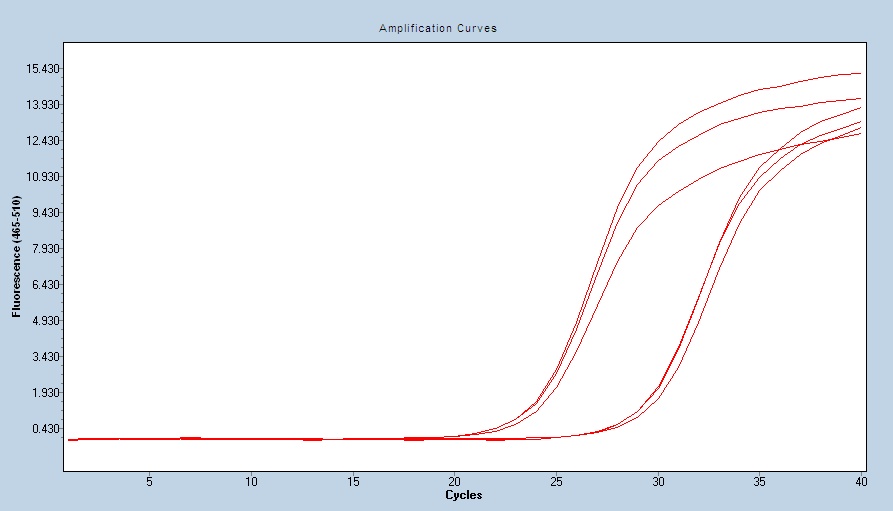

Supplement: Supplementary file 3 [file Data_Sheet_1.zip › raw date of qRT-PCR/2. Amplification Plot-qRT-PCR/ALKBH5.jpg]

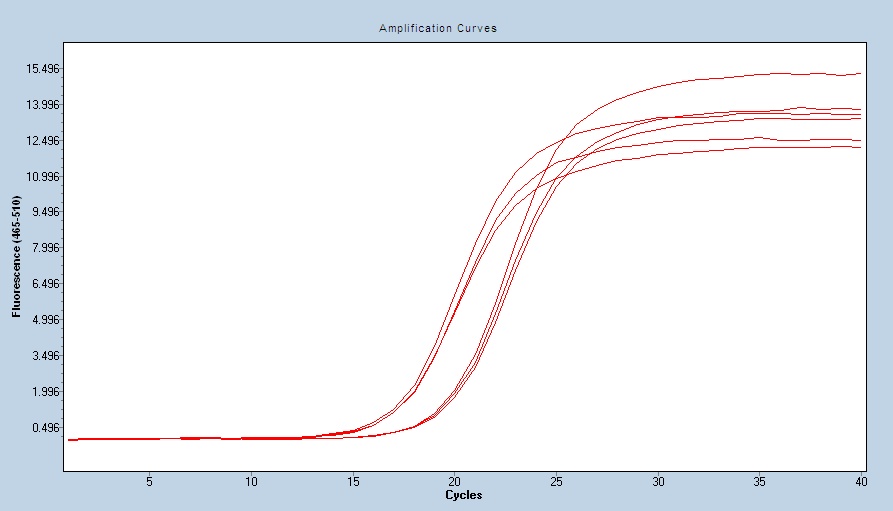

Supplement: Supplementary file 3 [file Data_Sheet_1.zip › raw date of qRT-PCR/2. Amplification Plot-qRT-PCR/GAPDH.jpg]

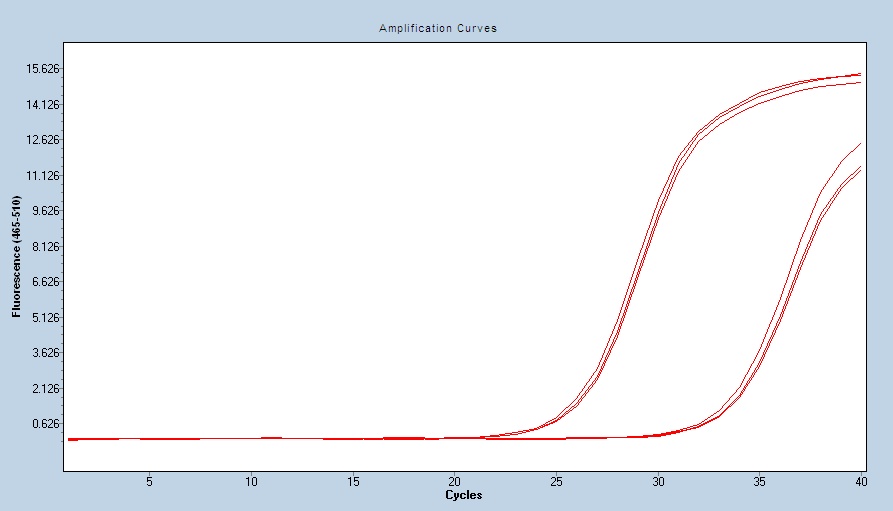

Supplement: Supplementary file 3 [file Data_Sheet_1.zip › raw date of qRT-PCR/2. Amplification Plot-qRT-PCR/METTL14.jpg]

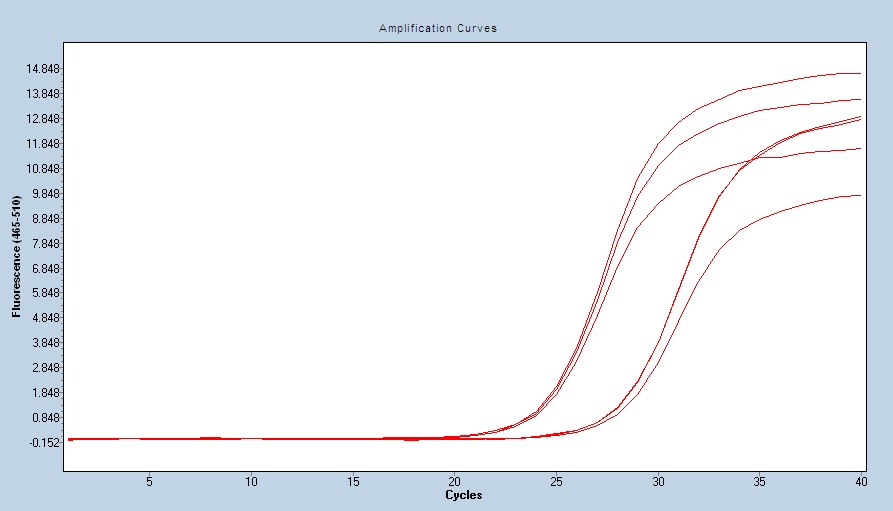

Supplement: Supplementary file 3 [file Data_Sheet_1.zip › raw date of qRT-PCR/2. Amplification Plot-qRT-PCR/METTL3.jpg]

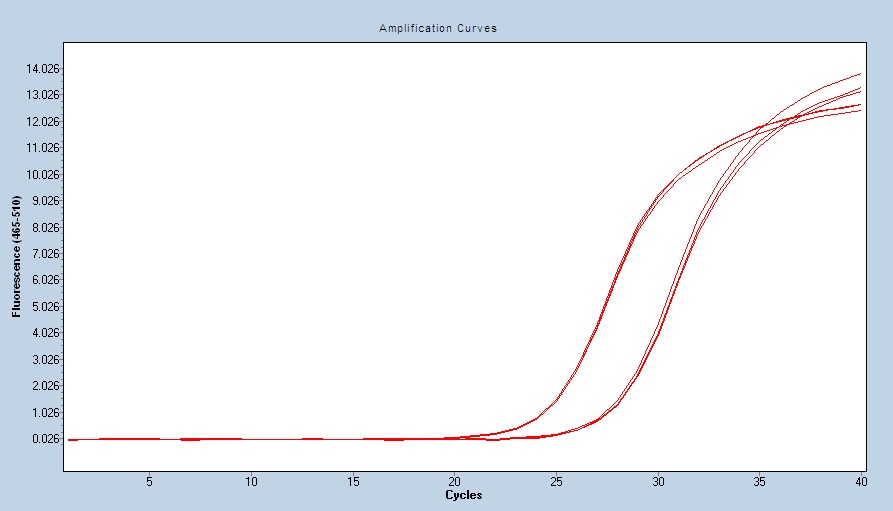

Supplement: Supplementary file 3 [file Data_Sheet_1.zip › raw date of qRT-PCR/2. Amplification Plot-qRT-PCR/WTAP.jpg]

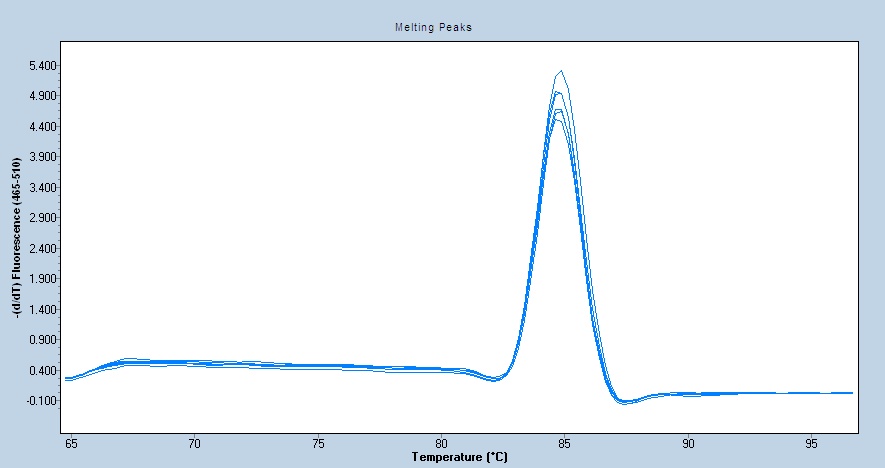

Supplement: Supplementary file 3 [file Data_Sheet_1.zip › raw date of qRT-PCR/3. Melt Curve Plot-qRT-PCR/ALKBH5.jpg]

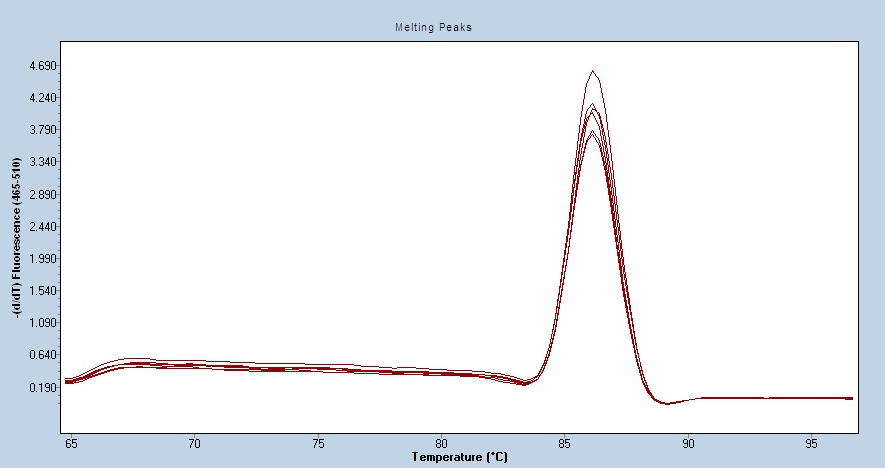

Supplement: Supplementary file 3 [file Data_Sheet_1.zip › raw date of qRT-PCR/3. Melt Curve Plot-qRT-PCR/GAPDH.jpg]

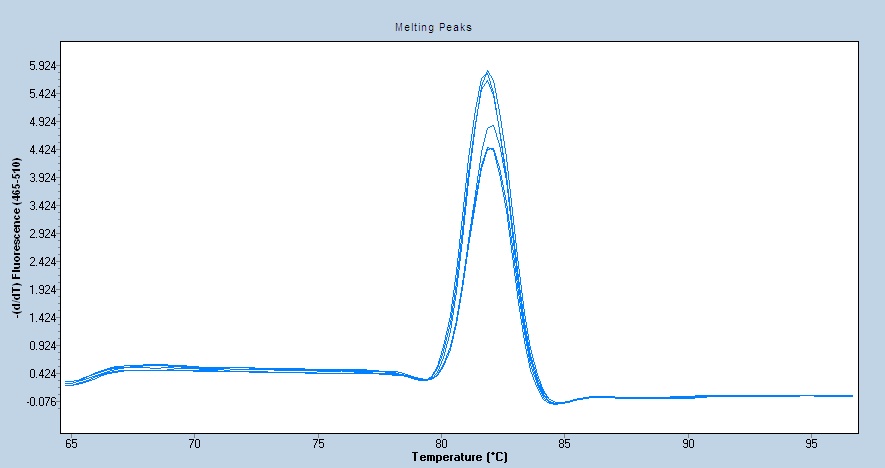

Supplement: Supplementary file 3 [file Data_Sheet_1.zip › raw date of qRT-PCR/3. Melt Curve Plot-qRT-PCR/METTL14.jpg]

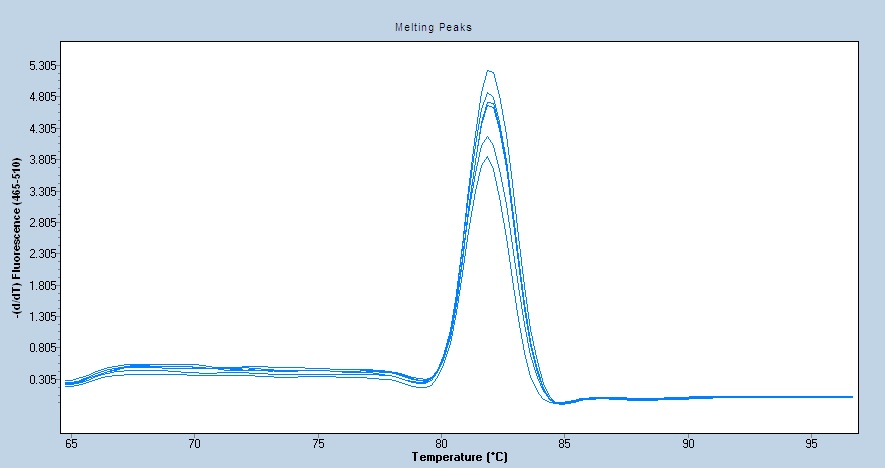

Supplement: Supplementary file 3 [file Data_Sheet_1.zip › raw date of qRT-PCR/3. Melt Curve Plot-qRT-PCR/METTL3.jpg]

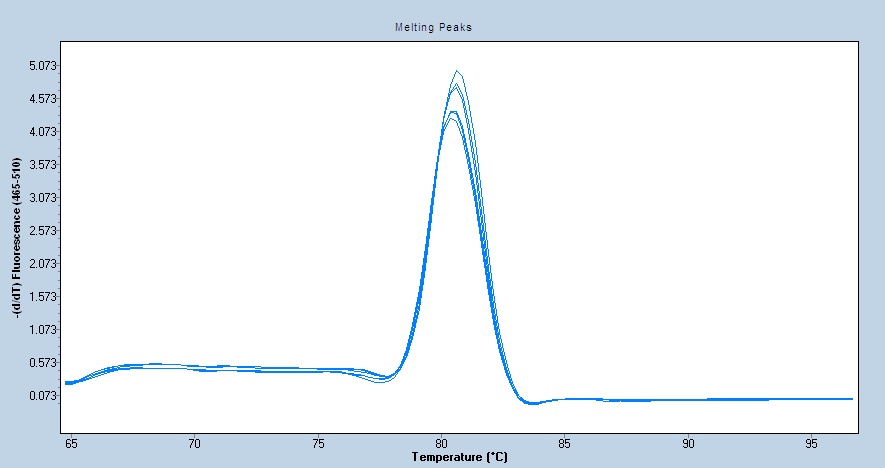

Supplement: Supplementary file 3 [file Data_Sheet_1.zip › raw date of qRT-PCR/3. Melt Curve Plot-qRT-PCR/WTAP.jpg]

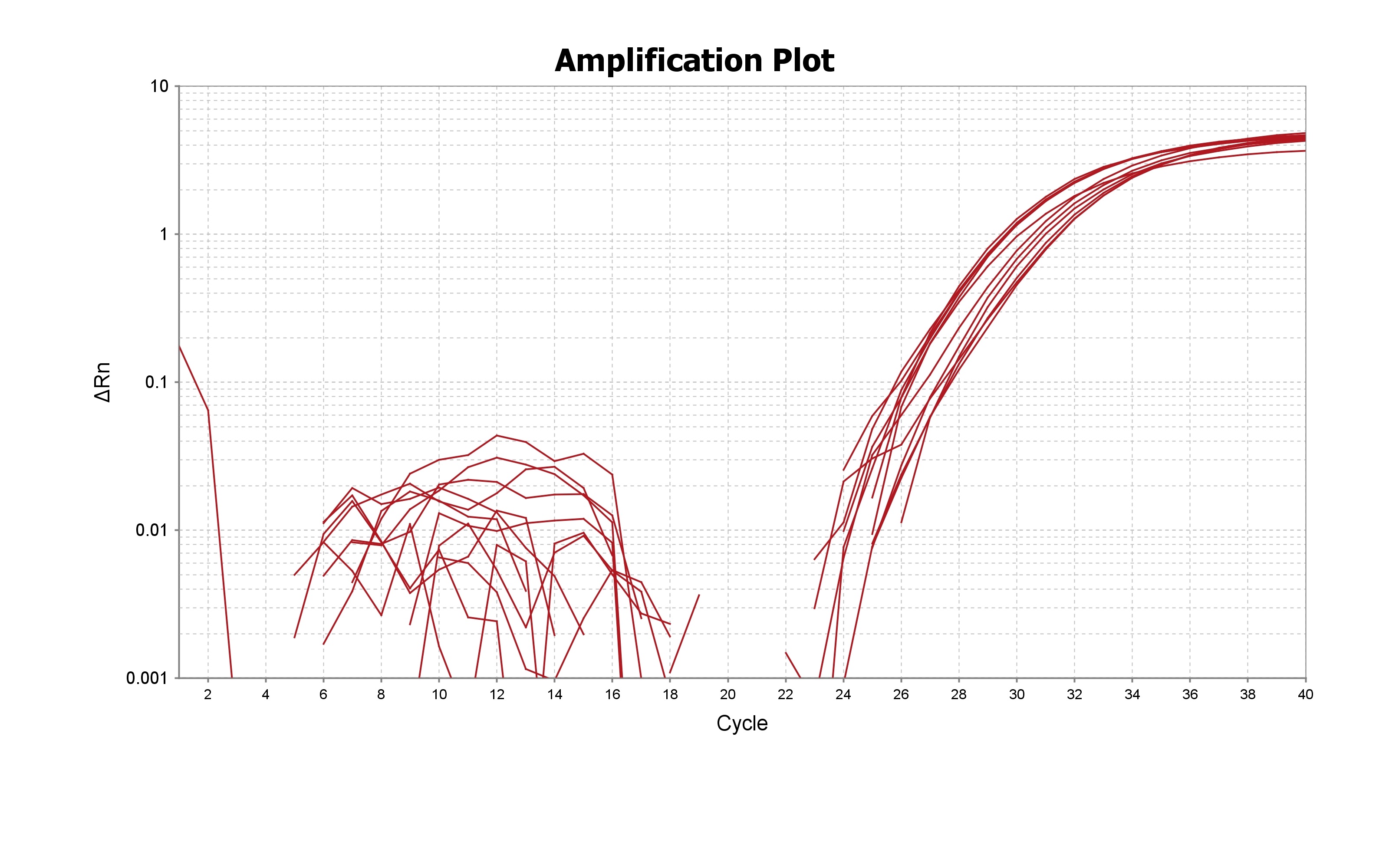

Supplement: Supplementary file 4 [file Data_Sheet_2.zip › raw date of Gene-specific m6A qPCR/2.Amplification Plot_merip-qpcr/Atg7-1.jpg]

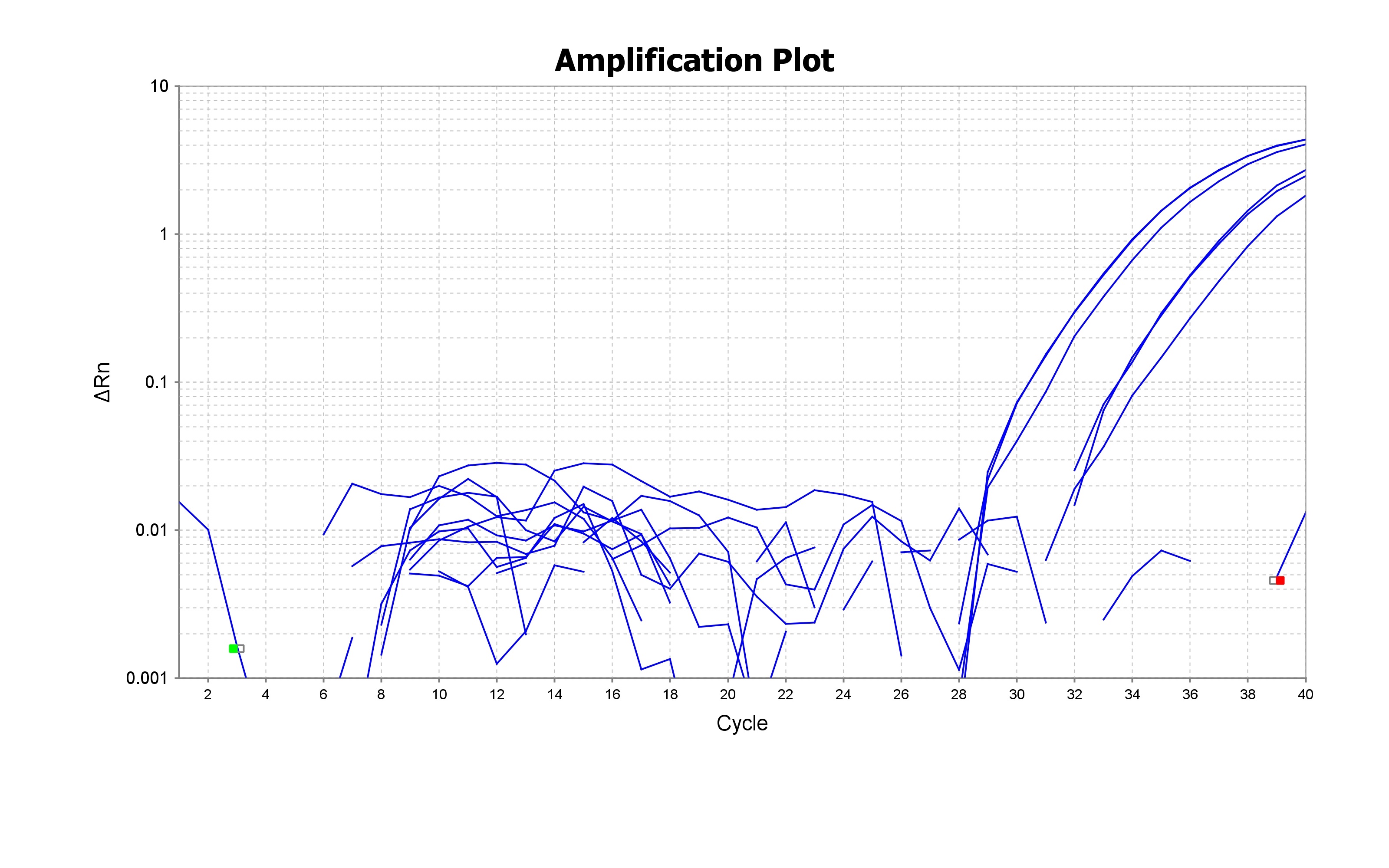

Supplement: Supplementary file 4 [file Data_Sheet_2.zip › raw date of Gene-specific m6A qPCR/2.Amplification Plot_merip-qpcr/Atg7-2.jpg]

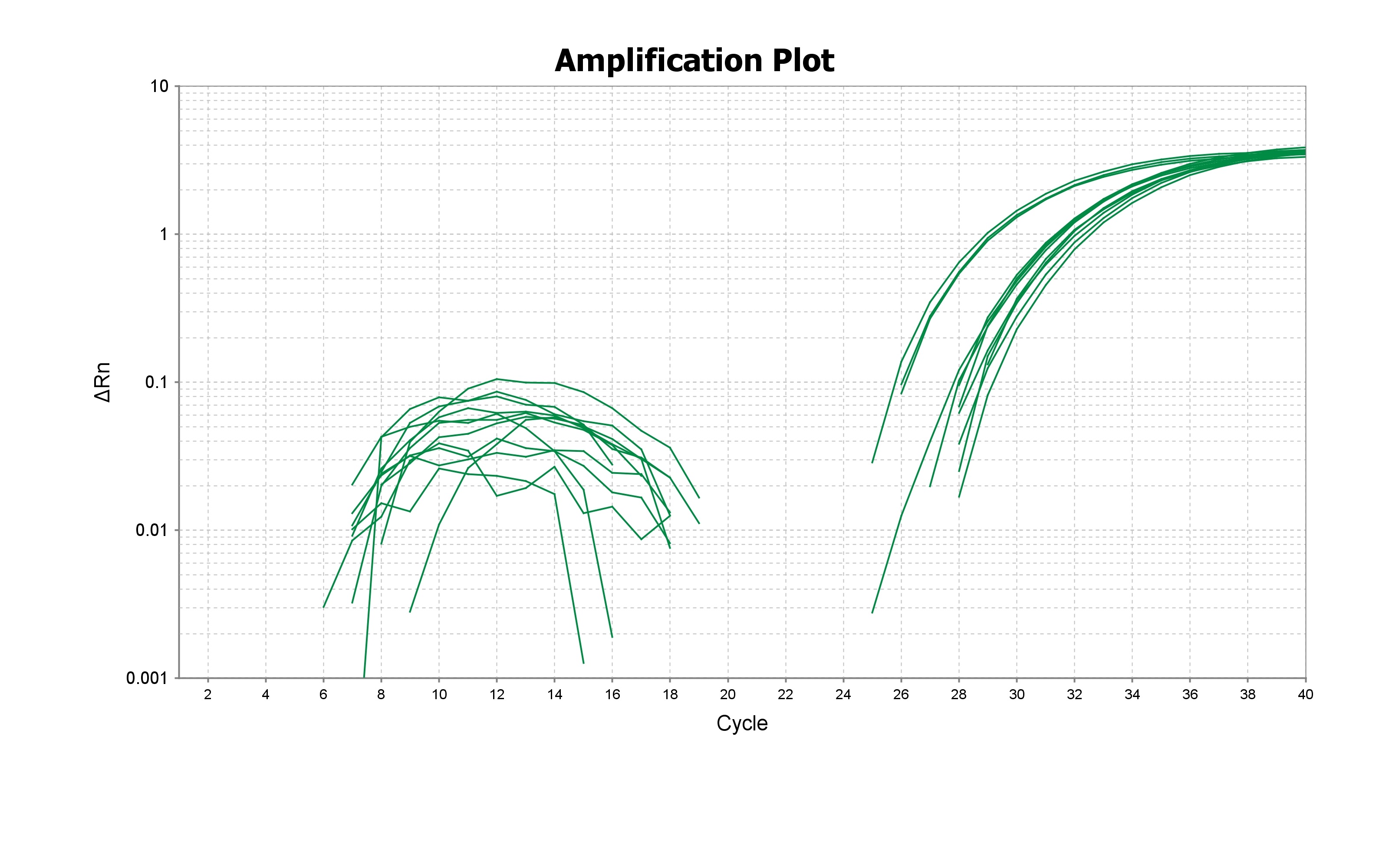

Supplement: Supplementary file 4 [file Data_Sheet_2.zip › raw date of Gene-specific m6A qPCR/2.Amplification Plot_merip-qpcr/Atg7-3.jpg]

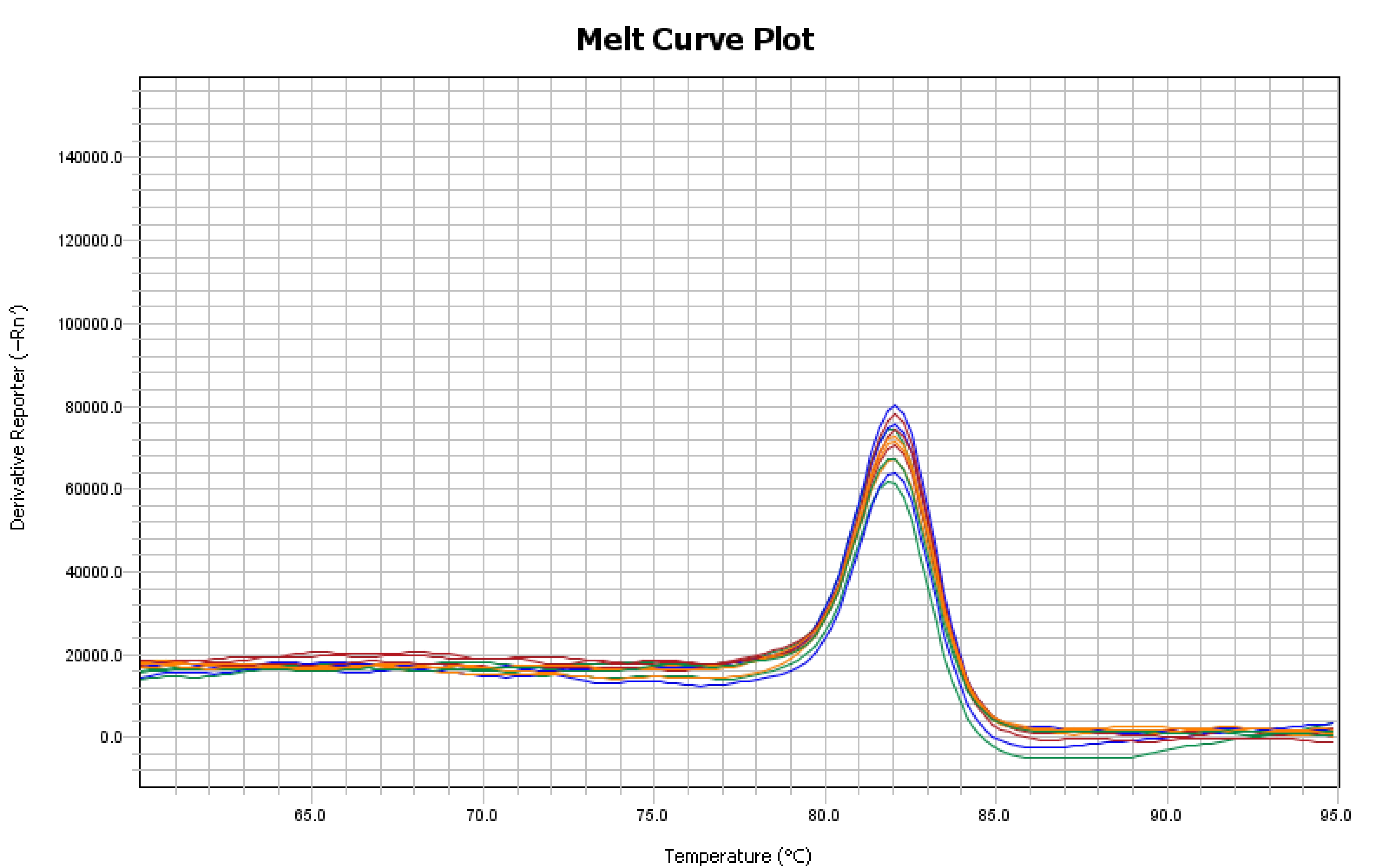

Supplement: Supplementary file 4 [file Data_Sheet_2.zip › raw date of Gene-specific m6A qPCR/3.Melt Curve Plot_merip-qpcr/Atg7-1.jpg]

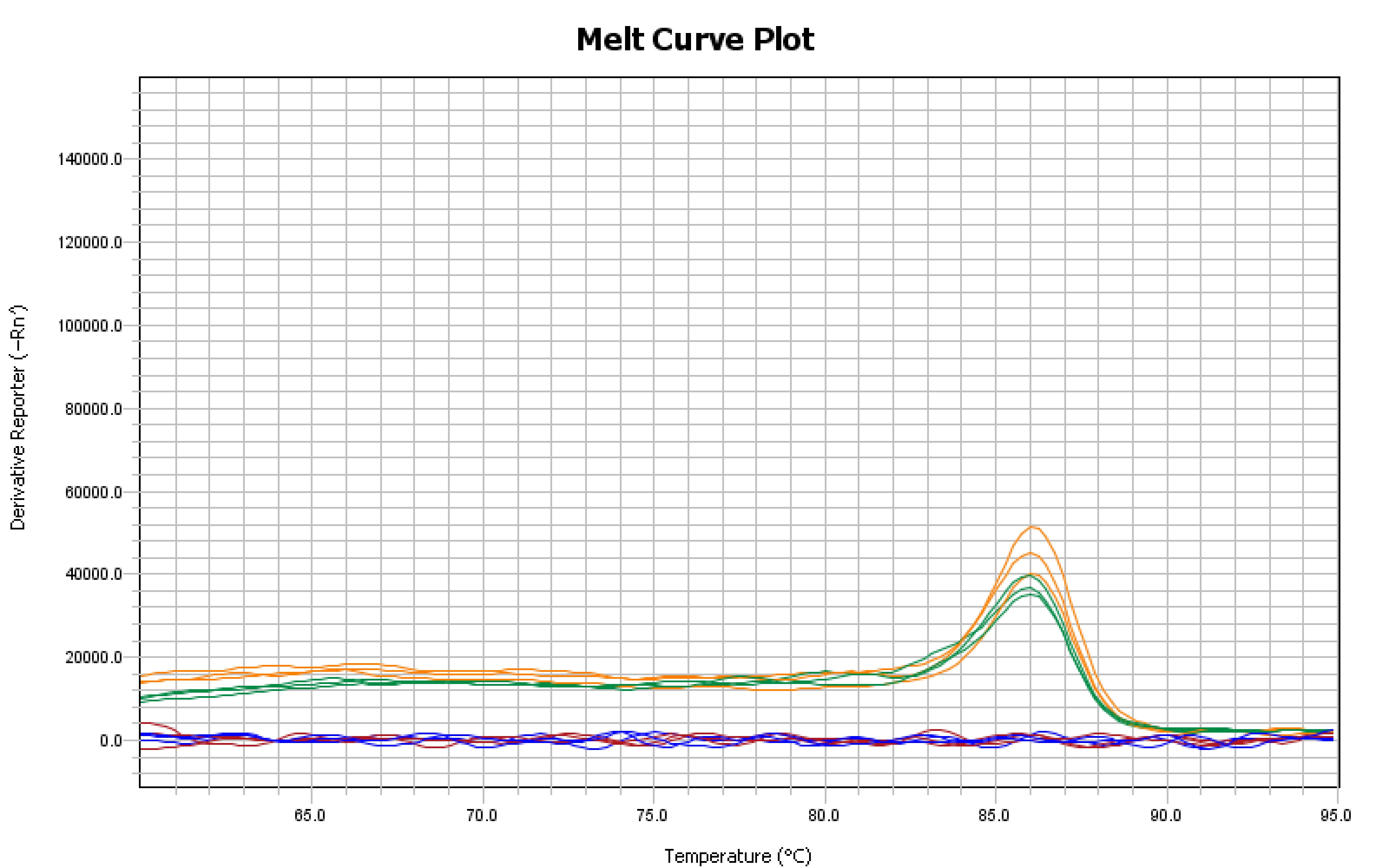

Supplement: Supplementary file 4 [file Data_Sheet_2.zip › raw date of Gene-specific m6A qPCR/3.Melt Curve Plot_merip-qpcr/Atg7-2.jpg]

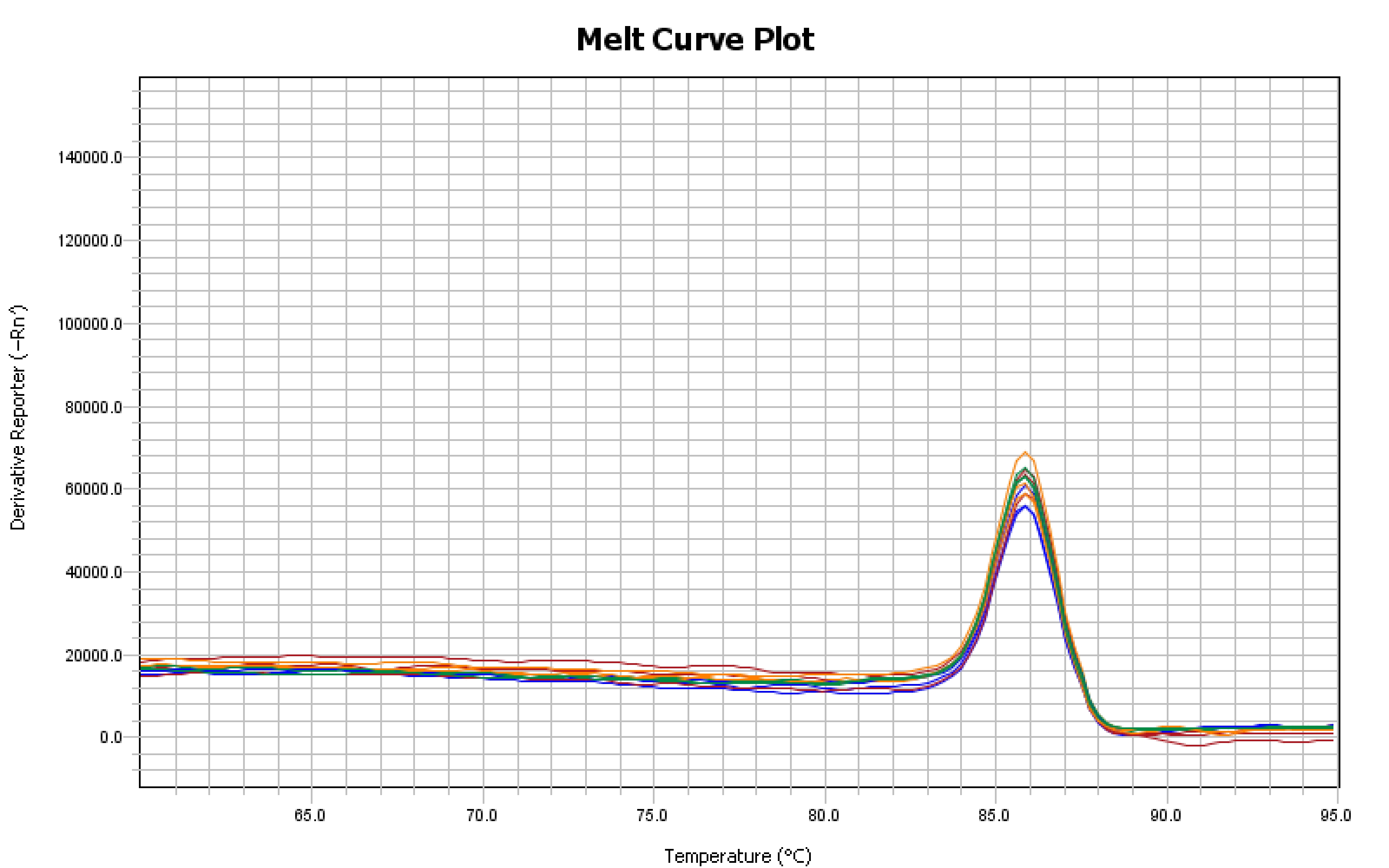

Supplement: Supplementary file 4 [file Data_Sheet_2.zip › raw date of Gene-specific m6A qPCR/3.Melt Curve Plot_merip-qpcr/Atg7-3.jpg]

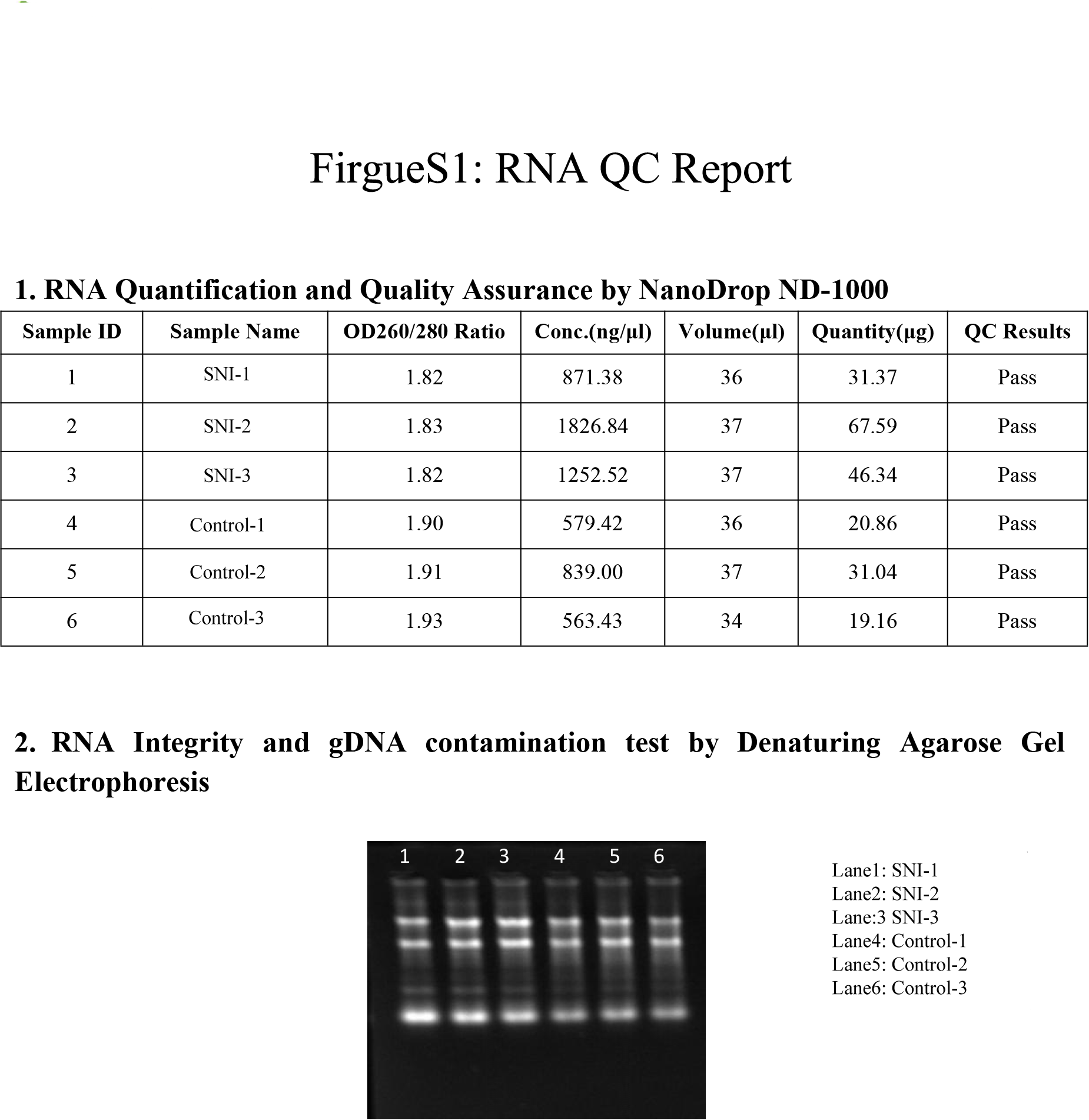

Supplement: Supplementary file 5 [file Image_1.TIF]
