# Supplementary material for: Epitranscriptomic Analysis of m6A Methylome After Peripheral Nerve Injury
Source: Front Genet. 2021 Jul 9;12:686000. doi: 10.3389/fgene.2021.686000 (PMC8301379; doi:10.3389/fgene.2021.686000)
Supplement: Supplementary file 6 [file Image_2.pdf]

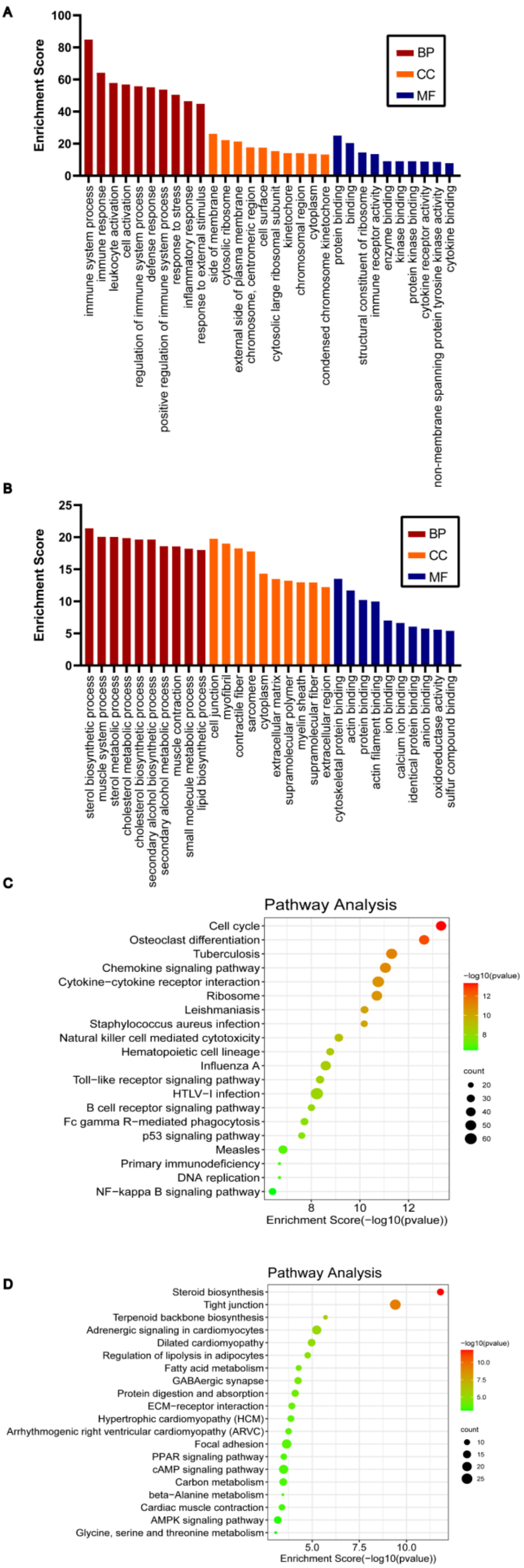
A
Enrichment Score
100
80
60
40
20
0
BP
CC
MF
immune system process
immune response
leukocyte activation
cell activation
regulation of immune system process
defense response
positive regulation of immune system process
response to stress
inflammatory response
response to external stimulus
side of membrane
cytosolic ribosome
external side of plasma membrane
chromosome, centromeric region
cell surface
cytosolic large ribosomal subunit
kinetochore
chromosomal region
cytoplasm
condensed chromosome kinetochore
protein binding
binding
structural constituent of ribosome
immune receptor activity
enzyme binding
kinase binding
protein kinase binding
cytokine receptor activity
non-membrane spanning protein tyrosine kinase activity
cytokine binding
B
Enrichment Score
25
20
15
10
5
0
BP
CC
MF
sterol biosynthetic process
muscle system process
sterol metabolic process
cholesterol metabolic process
cholesterol biosynthetic process
secondary alcohol biosynthetic process
secondary alcohol metabolic process
muscle contraction
small molecule metabolic process
lipid biosynthetic process
cell junction
myofibril
contractile fiber
sarcomere
cytoplasm
extracellular matrix
supramolecular polymer
myelin sheath
supramolecular fiber
extracellular region
cytoskeletal protein binding
actin binding
protein binding
actin filament binding
ion binding
calcium ion binding
identical protein binding
anion binding
oxidoreductase activity
sulfur compound binding
C
Pathway Analysis
Cell cycle
Osteoclast differentiation
Tuberculosis
Chemokine signaling pathway
Cytokine−cytokine receptor interaction
Ribosome
Leishmaniasis
Staphylococcus aureus infection
Natural killer cell mediated cytotoxicity
Hematopoietic cell lineage
Influenza A
Toll−like receptor signaling pathway
HTLV−I infection
B cell receptor signaling pathway
Fc gamma R−mediated phagocytosis
p53 signaling pathway
Measles
Primary immunodeficiency
DNA replication
NF−kappa B signaling pathway
8
10
12
Enrichment Score(−log10(pvalue))
−log10(pvalue)
12
10
8
count
20
30
40
50
60
D
Pathway Analysis
Steroid biosynthesis
Tight junction
Terpenoid backbone biosynthesis
Adrenergic signaling in cardiomyocytes
Dilated cardiomyopathy
Regulation of lipolysis in adipocytes
Fatty acid metabolism
GABAergic synapse
Protein digestion and absorption
ECM−receptor interaction
Hypertrophic cardiomyopathy (HCM)
Arrhythmogenic right ventricular cardiomyopathy (ARVC)
Focal adhesion
PPAR signaling pathway
cAMP signaling pathway
Carbon metabolism
beta−Alanine metabolism
Cardiac muscle contraction
AMPK signaling pathway
Glycine, serine and threonine metabolism
5.0
7.5
10.0
Enrichment Score(−log10(pvalue))
−log10(pvalue)
10.0
7.5
5.0
count
10
15
20
25

Figure S2: GO and KEGG pathway analyses of differential gene expression. Major gene ontology terms of the up-regulated (A) and down-regulated genes (B), respectively. Major enriched pathways of the up-regulated (C) and down-regulated (D)genes.
